# International Medical Congress

**Published:** 1887-03

**Authors:** 


INTERNATIONAL MEDICAL CONGRESS—*Section of Dental and Oral Surgery.*—Dr. E. A. Bogue, Secretary, 29 East 20th street, New York City, desires all who will assist in the Dental and Oral Section, to furnish him with an abstract of their papers, or the papers themselves, at once; as these papers must be in the hands of the Secretary as soon as possible, in order that a definite programme may be arranged, and a proper amount of time apportioned for each subject.

